# A multiplex dual-probe RT-LAMP assay for rapid subtype-specific detection of respiratory syncytial virus A and B

**DOI:** 10.1371/journal.pone.0354914

**Published:** 2026-07-31

**Authors:** Min Sup Lim, Chansoo Park, Eunji Lee, Sun-Young Ko, Woong Sik Jang

**Affiliations:** 1 Department of Health and Safety Convergence Science, Korea University, Seoul, Republic of Korea; 2 Department of Laboratory Medicine, College of Medicine, Korea University Guro Hospital, Guro-gu, Seoul, Republic of Korea; 3 Department of Biomedical Sciences, BK21 Graduate Program, College of Medicine, Korea University, Seongbuk-gu, Seoul, Republic of Korea; 4 Department of Laboratory Medicine, Korea University Ansan Hospital, Korea University College of Medicine, Seoul, Republic of Korea; 5 Emergency Medicine, College of Medicine, Korea University Guro Hospital, Guro-gu, Seoul, Republic of Korea; Cairo University Faculty of Veterinary Medicine, EGYPT

## Abstract

Respiratory syncytial virus (RSV) is a leading cause of acute respiratory tract infections, particularly in infants, older adults, and immunocompromised individuals. RSV is classified into two major subtypes, RSV A and RSV B, which co-circulate seasonally and exhibit genetic variability, highlighting the need for rapid and subtype-specific diagnostic methods. Although reverse transcription quantitative PCR (RT-qPCR) is the reference standard for RSV detection, its reliance on complex instrumentation limits its applicability in decentralized testing settings. In this study, we developed and evaluated a probe-based reverse transcription loop-mediated isothermal amplification (RT-LAMP) assay for rapid detection and differentiation of RSV A and RSV B. The assay incorporates a dual-probe strategy, employing an assimilating probe for RSV A detection to ensure robust signal generation under multiplex conditions, and hybridization-based TaqMan-style probes (HyTaq probes) for RSV B detection and for an internal control targeting the human ACTB gene to ensure reaction validity. Analytical performance was assessed using serially diluted RSV positive clinical specimens and plasmid standards. Clinical performance was evaluated using 91 RSV A positive specimens, 97 RSV B positive specimens, and 120 RSV negative specimens, as defined by the reference diagnosis. The RSV A and RSV B RT-LAMP assays demonstrated sensitivities of 92.31% and 98.97%, respectively, with a specificity of 100% for both targets. No cross-reactivity was observed with a panel of common respiratory viruses. These results indicate that the proposed dual-probe RT-LAMP assay provides a rapid and specific approach for subtype-specific RSV detection, with potential applicability in decentralized diagnostic settings pending further validation.

## Introduction

Respiratory syncytial virus (RSV) is a leading etiologic agent of acute lower respiratory tract infections worldwide and poses a substantial clinical burden, particularly among infants, young children, older adults, and immunocompromised individuals [1,2]. Global burden of disease studies estimate that RSV accounts for millions of hospitalizations and a considerable number of deaths each year in children under five years of age, emphasizing the persistent need for rapid and reliable diagnostic approaches [2,3]. RSV is classified into two major antigenic subtypes, RSV A and RSV B, which co‑circulate during seasonal epidemics and exhibit notable genetic variability, especially within the attachment (G) glycoprotein gene [4]. Epidemiological surveillance has demonstrated that the relative predominance of RSV A and RSV B varies across seasons and geographic regions, underscoring the importance of subtype‑resolved detection for molecular epidemiology, outbreak monitoring, and public health surveillance [4,5]. Furthermore, subtype‑specific information is increasingly relevant for tracking viral evolution in the context of expanding RSV immunization strategies and the clinical deployment of monoclonal antibody‑based interventions [1,3]. In particular, the recent approval of nirsevimab, a monoclonal antibody targeting the RSV fusion protein, and the licensure of mRNA-based RSV vaccines for older adults underscore the growing clinical relevance of subtype-resolved surveillance, as differential subtype prevalence may influence the effectiveness of these preventive interventions and inform future immunization policy [6].

Reverse transcription quantitative polymerase chain reaction (RT‑qPCR) is currently regarded as the reference standard for RSV detection because of its high analytical sensitivity and specificity, and subtype‑specific RT‑qPCR assays capable of discriminating between RSV A and RSV B have been established [7]. Despite these advantages, RT‑qPCR requires centralized laboratory infrastructure, sophisticated instrumentation, and skilled personnel, which collectively limit its utility in decentralized, resource‑limited, or time‑critical clinical settings. Loop‑mediated isothermal amplification (LAMP), first described by Notomi et al., enables rapid nucleic acid amplification under isothermal conditions and has been adapted for RNA virus detection by incorporating a reverse transcription step (RT‑LAMP) [8]. Owing to its minimal equipment requirements and short time‑to‑result, RT‑LAMP has emerged as a promising molecular diagnostic platform for point‑of‑care and near‑patient testing [9]. Multiple RT‑LAMP assays for RSV have been reported, including early studies demonstrating subgroup detection and subsequent work describing direct detection from clinical respiratory specimens [10]. In addition, multiplex RT‑LAMP assays combining primer sets for RSV A and RSV B within a single reaction have been described, demonstrating the technical feasibility of simultaneous isothermal amplification. However, these assays relied on shared fluorescence or non‑specific detection readouts and did not enable reliable discrimination between RSV A and RSV B [11]. Notably, the multiplex LAMP assay reported by Mahony et al. attempted subtype discrimination based on amplicon annealing temperature (Tm); however, overlapping Tm values between RSV A and RSV B precluded reliable subtype-specific identification within a single reaction.

Most previously reported RSV RT‑LAMP assays are based on primer‑driven amplification coupled with non‑sequence‑specific signal generation strategies, including intercalating dyes, turbidity measurement, or colorimetric indicators. Such detection approaches are inherently constrained by non‑specific amplification, a well‑recognized feature of LAMP chemistry, thereby limiting their suitability for precise analytical outputs such as subtype discrimination or multiplex interpretation [12]. To overcome these limitations, a variety of probe‑based detection strategies enabling sequence‑specific signal generation in LAMP reactions have been developed. These include assimilating probes that generate fluorescence upon hybridization with amplification products [13], molecular beacons that undergo conformational changes upon target binding [14], fluorescence resonance energy transfer (FRET)‑based probes [15], and TaqMan‑style hybridization‑based probes that yield target‑dependent fluorescence signals through hybridization and enzymatic reactions [16]. Collectively, these probe‑based approaches reduce non‑specific signal generation and facilitate reliable multiplex fluorescence readout in isothermal amplification assays [17].

In this study, we report the development of a probe‑based multiplex RT‑LAMP assay that enables simultaneous and subtype‑specific detection of RSV A and RSV B, together with an internal amplification control, within a single isothermal reaction. An assimilating probe strategy was employed for RSV A detection to ensure robust signal generation under multiplex conditions, whereas hybridization‑assisted TaqMan‑style probes (HyTaq probes) were designed and applied for RSV B detection and for an internal control targeting the human actin beta (ACTB) gene to enhance sequence specificity. We aimed to optimize reaction conditions and evaluate both analytical and clinical performance of the assay, with direct comparison against a commercial RT‑qPCR assay, to establish its utility as a rapid and specific tool for subtype-specific RSV detection.

## Materials and methods

### Clinical samples and DNA extraction

For clinical validation of the multiplex RSV A/B/IC LAMP assay, a total of 308 nasopharyngeal (NP) swab specimens were retrospectively obtained from Korea University Guro Hospital (Seoul, Republic of Korea). The specimens had been originally collected between January 2018 and July 2019 as part of routine clinical care and consisted of 91 RSV A–positive, 97 RSV B–positive, and 120 RSV negative samples, as determined during routine clinical diagnostics using the Allplex Respiratory Panel 1 assay (Seegene, Seoul, Republic of Korea). These prior diagnostic results were used as the reference standard for performance evaluation, reflecting routine clinical diagnostic practice, and were defined independently of the multiplex RSV A/B/IC LAMP assay evaluated in this study. For comparative analysis, a commercial RT-PCR assay, Allplex SARS-CoV-2/FluA/FluB/RSV Assay (Seegene, Seoul, Republic of Korea) was also tested using the same specimen set. Total nucleic acids were extracted from all NP swab specimens using the PowerEXP™ 32 automated extraction system (KogeneBiotech, Seoul, Republic of Korea) according to the manufacturer’s instructions. Briefly, 200 µL of each clinical sample was loaded into a 96-well extraction plate and processed using the automated protocol. The extracted nucleic acids were stored at −20 °C until use in the LAMP assays and reference RT-PCR testing.

### Ethical statement

This retrospective study used residual clinical specimens obtained during routine diagnostic testing. The study was conducted in accordance with the Declaration of Helsinki and approved by the Institutional Review Board of Korea University Guro Hospital (approval number: 2019GR0055), with an exemption from review and a waiver of informed consent. All samples were anonymized prior to analysis, and no identifiable information was used. The data were accessed for research purposes between September 1, 2025 and January 31, 2026.

### Multiplex primer and probe design

Primer sequences for RSV B and the internal control were designed using PrimerExplorer V5 (Eiken Chemical Co., Ltd.; https://primerexplorer.jp/lampv5e/) based on conserved regions of the target genes. The RSV B LAMP primer set was designed within a conserved region of the RNA-dependent RNA polymerase (L) gene of respiratory syncytial virus B. The RSV A LAMP primer set, targeting a conserved region of the matrix (M) gene of respiratory syncytial virus A, was adopted from a previously published multiplex LAMP assay [11]. The internal control (IC) LAMP primer set targeting the human actin beta (ACTB) gene was identical to that previously described by our study group and was included as an internal amplification control to monitor reaction validity [18]. For multiplex fluorescence detection, different probe chemistries were employed depending on the target. An assimilating probe was used for detection of RSV A, whereas HyTaq probes were used for detection of RSV B and the IC. The RSV A assimilating probe was labeled with FAM, while the RSV B and IC HyTaq probes were labeled with Cy5 and Hex, respectively. Corresponding quenchers were incorporated into each probe design to enable target-specific fluorescence signal generation during amplification. All LAMP primers and probes were synthesized by Macrogen Inc. (Seoul, Republic of Korea), and the detailed sequences and concentrations are provided in Table 1.

**Table 1:** RSV A/B/IC LAMP primer sets used in this study.

Primer Mix	Target Gene	Name	Sequence (5’-3’)	μM
RSV A LAMP primer Mix	Matrix (M) gene	F3	GCTGTTCAATACAATGTCCTAGA	4
		B3	GGTAAATTTGCTGGGCATT	4
		FIP	TCTGCTGGCATGGATGATTGG-AGACGATGATCCTGCATCA	32
		BIP	CTAGTGAAACAAATATCCACACCCA-GCACTGCACTTCTTGAGTT	32
		LF	ACATGGGCACCCATATTGTAAG	10
		LB	AGGGACCTTCATTAAGAGTCATGAT	4
		RSV A probe	FAM-CGG GCC CGT ACA AAG GGA ACA CCC ACA CTC CGA GGG ACC TTC ATT AAG AGT CAT GAT	6
			GAG TGT GGG TGT TCC CTT TGT ACG GGC CCG	9
RSV B LAMP primer Mix	RNA-dependent RNA polymerase (L) gene	F3	TTTGGTGGTGGTGATCCT	4
		B3	CAAACTCGGCATTTGGATT	4
		FIP	AGCTCAACACAAACACTGAATGTAC-TATATCGAAGCTTTTATAGGAGAAC	32
		BIP	CTGGTCACGATCTACAAGATAAGC-TGTGATGACACAAGTCAAGAA	32
		LF	TATAGCTTCTGTAAGGAAGTCTGGA	10
		LB	GGATCTTCCAGATGATAGACTGAAC	4
		RSV B probe	Cy5-GGATCTTCCAGATGATAGACTGAAC-BHQ2	10
Internal Control (IC) LAMP primer Mix	Actin beta gene	F3	AGT ACC CCA TCG AGC ACG	4
		B3	AGC CTG GAT AGC AAC GTA CA	4
		FIP	GAG CCA CAC GCA GCT CAT TGT ATC ACC AAC TGG GAC GAC A	32
		BIP	CTG AAC CCC AAG GCC AAC CGG CTG GGG TGT TGA AGG TC	32
		LF	TGT GGT GCC AGA TTT TCT CCA	10
		LB	CGA GAA GAT GAC CCA GAT CAT GT	4
		Actin B probe	Hex-CGAGAAGATGACCCaGATCATGT-BHQ1	10

### Multiplex RSV A/B/IC LAMP assay

The multiplex RSV A/B/IC LAMP assay was performed using ELPIS RT-LAMP 2X Master Mix (Elpis-Biotech, Daejeon, Republic of Korea). The reaction mixture was prepared with 12.5 μL of 2X Master Mix, 6.5 μL of reaction-free water, 1 μL of RSV A LAMP primer mix, 1 μL of RSV B LAMP primer mix, and 1 μL of internal control (IC; actin beta) LAMP primer mix. Subsequently, 3 μL of extracted nucleic acid template was added, resulting in a final reaction volume of 25 μL. Each RSV A, RSV B, and IC LAMP primer mix contained 4 μM of each outer primer (F3 and B3), 32 μM of each inner primer (FIP and BIP), 10 μM of the loop forward (LF) primer, and 4 μM of the loop backward (LB) primer. For fluorescence detection, the RSV A primer mix included a fluorescent probe at a final concentration of 6 μM together with a complementary quencher oligonucleotide at 9 μM. The RSV B and IC primer mixes contained HyTaq probes at final concentrations of 10 μM each. Detailed primer and probe sequences and concentrations are provided in Table 1. All LAMP reactions were carried out using a CFX96 Touch Real-Time PCR Detection System (Bio-Rad Laboratories, Hercules, CA, USA) at 62 °C for 40 min, with real-time fluorescence signal acquisition.

### Comparative RT-PCR assay

For comparative evaluation, the performance of the multiplex RSV A/B/IC LAMP assay was assessed against a commercial real-time RT-PCR assay, the Allplex™ SARS-CoV-2/FluA/FluB/RSV Assay (Seegene, Seoul, Republic of Korea). RT-PCR amplification and fluorescence detection were carried out using a CFX96 Touch Real-Time PCR Detection System (Bio-Rad Laboratories, Hercules, CA, USA). All RT-PCR reactions were prepared and conducted in accordance with the manufacturer’s instructions. The thermal cycling conditions for the Allplex™ SARS-CoV-2/FluA/FluB/RSV assay consisted of an initial reverse transcription step at 50 °C for 20 min, followed by enzyme activation at 95 °C for 15 min. This was followed by two preliminary amplification cycles of 95 °C for 10 s, 60 °C for 40 s, and 72 °C for 20 s, and subsequently 41 amplification cycles of 95 °C for 10 s, 60 °C for 15 s, and 72 °C for 10 s. Fluorescence signals were acquired at the 60 °C and 72 °C steps according to the assay protocol.

### Analytical sensitivity assessment

The analytical sensitivity of the developed RSV A/B/IC LAMP assay was assessed using plasmid DNA standards and serially diluted clinical specimens. Plasmids containing the target regions of RSV A and RSV B were separately constructed by cloning PCR-amplified target sequences into pMG-Amp cloning vectors (Macrogen Inc., Seoul, Republic of Korea). Plasmid DNA concentrations were determined by Macrogen Inc., and copy numbers were calculated using an online copy number calculator (Copy Number Calculator, Technology Networks; https://www.technologynetworks.com/genomics/tools/copy-number-calculator). The plasmids were serially diluted 10-fold to generate concentrations corresponding to 10^0^–10^7^ copies per reaction, with nuclease-free water included as a no-template control. An aliquot of each dilution (1 µL) was added to the LAMP reaction mixture and amplified under identical conditions, and each concentration was tested in triplicate. The lowest concentration that yielded positive amplification in all replicates (3/3) was regarded as the lowest detectable level under the tested conditions. To directly compare the analytical sensitivity of the developed LAMP assay with that of a commercial RT-PCR assay, nasopharyngeal swab samples previously confirmed as RSV A– or RSV B–positive were serially diluted 10-fold using viral transport medium or nuclease-free water to generate a dilution series from undiluted to 10^−6^. Each dilution was tested in parallel using the multiplex RSV A/B/IC LAMP assay and the Allplex™ SARS-CoV-2/FluA/FluB/RSV Assay (Seegene Inc., Seoul, South Korea), according to the manufacturer’s instructions. Since the two assays target different genomic regions of RSV, direct conversion of dilution-based results to absolute viral copy numbers was not feasible. Therefore, relative analytical sensitivity was compared based on the lowest detectable dilution yielding consistent positive amplification across all replicates (3/3). All reactions were performed in triplicate, and the highest dilution level that met the same positivity criterion was considered the lowest detectable dilution for each assay. Cycle threshold (Ct) or time to threshold (Tt) values were recorded, and mean values and standard deviations were calculated for each dilution level, while samples showing no amplification were recorded as not detected (N/D).

### Cross-reactivity testing

To evaluate cross-reactivity, a panel of respiratory pathogen–positive clinical specimens was included. The panel comprised one sample positive for each of the following 12 pathogens: human coronavirus 229E, coronavirus NL63, coronavirus OC43, influenza A (H1N1), influenza A (H3N2), influenza B, human enterovirus (HEV), adenovirus (AdV), parainfluenza virus (PIV), human metapneumovirus (hMPV), human bocavirus (HBoV), and human rhinovirus (HRV). In addition, clinical specimens positive for *Mycoplasma pneumoniae* and *Bordetella pertussis* were included, along with cultured isolates of *Streptococcus pneumoniae, Staphylococcus aureus*, and *Klebsiella pneumoniae*. All viral and bacterial respiratory pathogens were confirmed by routine molecular diagnostic testing performed at the Department of Laboratory Medicine, Korea University Guro Hospital, independently of the LAMP assay. Each sample was tested in triplicate using the multiplex RSV A/B/IC LAMP assay under the same conditions described above.

### Statistical analysis

Sensitivity, specificity, and standard deviations (SDs) were calculated using Microsoft Excel (Microsoft Corporation, Redmond, WA, USA). The 95% confidence intervals (CIs) for sensitivity and specificity were calculated using the Wilson score interval method. Sensitivity was defined as the proportion of reference-positive specimens correctly identified by the LAMP assay, and specificity was defined as the proportion of reference-negative specimens correctly identified. Sensitivity, specificity, PPV, NPV, accuracy and Cohen’s κ were calculated relative to the reference diagnosis established by the Allplex Respiratory Panel 1 assay. Cohen’s κ coefficient was calculated from the 2 × 2 contingency table using the standard formula, κ = (Po − Pe)/ (1 − Pe), where Po is the observed agreement and Pe is the expected agreement by chance. For descriptive comparison, the Allplex™ SARS-CoV-2/FluA/FluB/RSV Assay was tested in parallel using the same specimen set, and results are presented in Table 4. All statistical analyses were performed using Microsoft Excel.

## Results

### Optimization of the multiplex RSV A/B/IC LAMP assay

**Fig 1:**
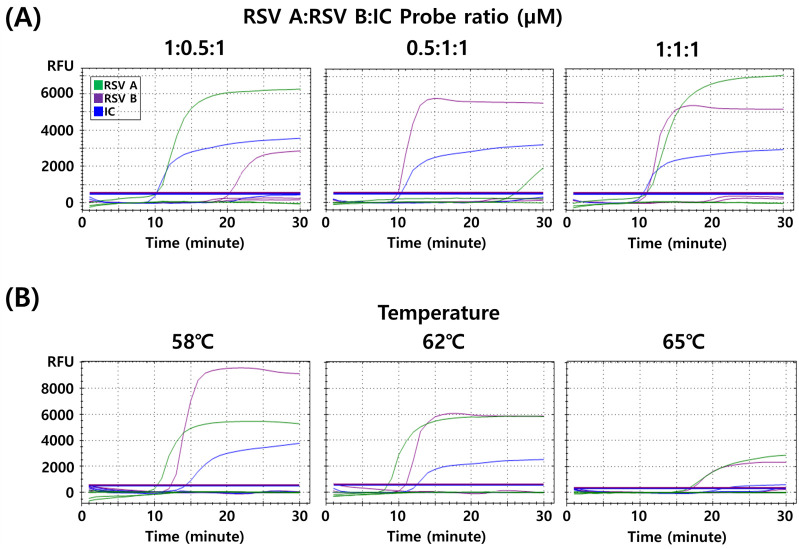
Optimization of probe ratio and reaction temperature for the multiplex RSV A/B/IC LAMP assay. (A) Optimization of probe concentration ratios for multiplex detection. Real-time amplification curves were obtained using RSV A and RSV B plasmid templates together with the internal control (IC) at 62 °C. Three different probe concentration ratios of RSV A, RSV B, and IC (1:0.5:1, 0.5:1:1, and 1:1:1) were evaluated. Fluorescence signals were monitored in the FAM channel (green) for RSV A, the Cy5 channel (purple) for RSV B, and the HEX channel (blue) for the internal control. (B) Optimization of reaction temperature for the multiplex RSV A/B/IC LAMP assay. Using the selected probe ratio, amplification reactions were performed at 58 °C, 62 °C, and 65 °C. The x-axis represents time to threshold (minutes) and the y-axis represents relative fluorescence units (RFU).

Optimization of the probe ratio and reaction temperature for the multiplex RSV A/B/IC LAMP assay was performed using RSV A and RSV B plasmid templates together with the internal control (IC). First, three different probe concentration ratios for RSV A, RSV B, and IC (1:0.5:1, 0.5:1:1, and 1:1:1) were evaluated at 62 °C (Fig 1A). At a ratio of 1:0.5:1, the onset of amplification for RSV B was delayed compared with RSV A and IC. At a ratio of 0.5:1:1, delayed amplification of RSV A was observed, accompanied by a lower fluorescence signal intensity relative to the other targets. In contrast, at a ratio of 1:1:1, RSV A, RSV B, and IC exhibited similar amplification onset times with stable, target-specific fluorescence signals. Based on these results, the probe ratio of 1:1:1 was selected for subsequent experiments. Next, reaction temperature was evaluated at 58 °C, 62 °C, and 65 °C using the selected probe ratio of 1:1:1 (Fig 1B). At 58 °C, amplification was detected with relatively delayed Tt values, with RSV A, RSV B, and IC detected at 16.68, 17.19, and 21.52 min, respectively. At 62 °C, Tt values were markedly reduced across all targets, with RSV A, RSV B, and IC detected at 8.53, 11.01, and 12.46 min, respectively. At 65 °C, RSV A, RSV B, and IC were detected at 10.37, 12.24, and 14.16 min, respectively. Based on these findings, a probe ratio of 1:1:1 (RSV A:RSV B:IC) and a reaction temperature of 62 °C were selected as the optimal conditions for the multiplex RSV A/B/IC LAMP assay.

### Analytical sensitivity of the multiplex RSV A/B/IC LAMP assay using plasmid DNA standards and clinical samples

The analytical sensitivity of the multiplex RSV A/B/IC LAMP assay was first evaluated using plasmid DNA standards containing the target sequences of RSV A and RSV B. Both RSV A and RSV B plasmids were consistently detected down to 1 × 10^4^ copies/reaction in all replicates (n = 3). At 1 × 10^3^ copies/reaction, amplification was not reproducibly observed and was absent at lower concentrations. Signals were detected exclusively in the corresponding fluorescence channels (FAM for RSV A and Cy5 for RSV B), and no cross-reactivity was observed between targets. The internal control (IC, Hex channel) was not amplified in plasmid-only reactions, as expected, confirming target-specific detection in the multiplex assay format (Table 2). Analytical sensitivity was further assessed using serially diluted RSV positive clinical specimens and compared with a commercial multiplex real-time RT-PCR assay, the Allplex™ SARS-CoV-2/FluA/FluB/RSV Assay (Seegene Inc., Seoul, South Korea). For RSV A clinical samples, the LAMP assay detected viral RNA up to the 10^−3^ dilution, whereas the commercial RT-PCR assay detected RSV A up to the 10^−4^ dilution. For RSV B clinical samples, the LAMP assay showed positive amplification up to the 10^−4^ dilution, while the RT-PCR assay detected RSV B up to the 10^−5^ dilution (Table 3). In all clinical sample experiments, amplification signals were observed only in the appropriate target-specific channels, and no false-positive signals were detected in non-target channels, indicating high assay specificity under multiplex conditions. These results demonstrate that although the commercial RT-PCR assay exhibited approximately one-log higher analytical sensitivity in diluted clinical samples, the multiplex RSV A/B/IC LAMP assay achieved reliable detection within clinically relevant concentration ranges. The rapid amplification and simplified isothermal operation of the assay suggest potential applicability in point-of-care settings, although evaluation with portable isothermal platforms remains necessary.

**Table 2:** Analytical sensitivity of the multiplex RSV A/B/IC LAMP assay using plasmid DNA standards.

Copies/reaction	Multiplex RSV A/B/IC LAMP assay					
	RSV A plasmid (Tt ± SD)			RSV B plasmid (Tt ± SD)		
	RSV A (FAM)	RSV B (Cy5)	IC (Hex)	RSV A (FAM)	RSV B (Cy5)	IC (Hex)
1×10^7^	9.95 ± 0.21	N/D	N/D	9.30 ± 0.01	N/D	N/D
1×10^6^	11.13 ± 0.22	N/D	N/D	10.64 ± 0.26	N/D	N/D
1×10^5^	13.30 ± 0.18	N/D	N/D	11.85 ± 0.20	N/D	N/D
1×10^4^	17.02 ± 2.95	N/D	N/D	14.64 ± 1.49	N/D	N/D
1×10^3^	N/D	N/D	N/D	N/D	N/D	N/D
1×10^2^	N/D	N/D	N/D	N/D	N/D	N/D
1×10^1^	N/D	N/D	N/D	N/D	N/D	N/D
1×10^0^	N/D	N/D	N/D	N/D	N/D	N/D
DW	N/D	N/D	N/D	N/D	N/D	N/D

Tt, time to threshold (minutes); SD, standard deviation; N/D, not detected; DW, distilled water.

**Table 3:** Comparison of analytical sensitivity between the multiplex RSV A/B/IC LAMP assay and the Allplex™ SARS-CoV-2/FluA/FluB/RSV Assay using diluted clinical samples.

Dilution	RSV A clinical sample				RSV B clinical sample			
	Seegene Assay (Ct ± SD)	Multiplex RSV A/B/IC LAMP assay (Tt ± SD)			Seegene Assay (Ct ± SD)	Multiplex RSV A/B/IC LAMP assay (Tt ± SD)		
		RSV A (FAM)	RSV B (Cy5)	IC (Hex)		RSV A (FAM)	RSV B (Cy5)	IC (Hex)
1×10^0^	21.43± 0.09	12.40± 0.56	N/D	12.48± 0.59	19.36± 0.45	N/D	11.11± 0.10	13.76± 0.74
1×10^−1^	24.11± 0.36	12.08± 0.52	N/D	13.20± 0.64	20.21± 0.78	N/D	12.25± 0.08	13.8± 0.12
1×10^−2^	30.59± 0.52	14.79± 0.39	N/D	15.10± 0.95	23.47± 0.59	N/D	13.99± 0.24	15.40± 0.97
1×10^−3^	35.20± 0.59	17.30± 1.87	N/D	N/D	27.69 ± 1.00	N/D	16.12± 0.36	21.37± 3.76
1×10^−4^	35.72± 0.62	N/D	N/D	N/D	31.74 ± 1.37	N/D	19.11± 1.17	20.82± 3.04
1×10^−5^	N/D	N/D	N/D	N/D	35.63± 0.13	N/D	N/D	N/D
1×10^−6^	N/D	N/D	N/D	N/D	N/D	N/D	N/D	N/D
DW	N/D	N/D	N/D	N/D	N/D	N/D	N/D	N/D

Ct, cycle threshold; Tt, time to threshold (minutes); SD, standard deviation; N/D, not detected; DW, distilled water.

**Table 4:** Clinical performance of the multiplex RSV A/B/IC LAMP assay and a commercial RT-PCR assay.

Clinical samples		Allplex SARS-CoV-2/FluA/FluB/RSV Assay	Multiplex RSV A/B/IC LAMP assay		
			RSV A (FAM)	RSV B (Cy5)	IC (Hex)
*RSV A* (n = 91)	P/Total	86/91	84/91	0/91	90/91
	Sensitivity (95% CI)	94.51% (87.7-97.6%)	92.31% (85.0–96.8%)	–	98.90% (94.0–99.8%)
	Specificity (95% CI)	–	–	100% (96.3–100)	–
*RSV B* (n = 97)	P/Total	96/97	0/97	96/97	96/97
	Sensitivity (95% CI)	98.97% (94.0–99.8%)	–	98.97% (94.0–99.8%)	98.97% (94.0–99.8%)
	Specificity (95% CI)	–	100% (96.3–100)	–	–
Non-Infected (n = 120)	P/Total	0/120	0/120	0/120	119/120
	Sensitivity (95% CI)	–	–	–	99.17% (95.4–99.9)
	Specificity (95% CI)	100% (96.9-100)	100% (96.9–100)	100% (96.9–100)	–

P, positive; CI, confidence interval; –, not applicable.

### Clinical performance of the multiplex RSV A/B/IC LAMP assay

The clinical performance of the multiplex RSV A/B/IC LAMP assay was evaluated in comparison with the Allplex SARS-CoV-2/FluA/FluB/RSV Assay (Seegene, Seoul, Republic of Korea) using 308 nasopharyngeal swab specimens, including 91 RSV A positive samples, 97 RSV B positive samples, and 120 RSV negative samples, as defined by the reference diagnosis (Table 4). For RSV A positive samples, the LAMP assay showed a sensitivity of 92.31% (84/91; 95% CI, 85.0–96.8), compared with 94.51% (86/91) for the Allplex SARS-CoV-2/FluA/FluB/RSV Assay (Table 4). No cross-reactivity was observed with the RSV B target for either assay, corresponding to a specificity of 100%. The internal control (IC) of the LAMP assay showed a positivity rate of 98.90% (90/91). For RSV B positive samples, both the LAMP assay and the Allplex SARS-CoV-2/FluA/FluB/RSV Assay demonstrated identical sensitivity of 98.97% (96/97), with no false-positive amplification observed for the RSV A target, yielding a specificity of 100% for both assays (Table 4). The IC positivity rate was 98.97% (96/97). Among RSV negative samples, no false-positive amplification was observed for either RSV A or RSV B targets in both assays, indicating a specificity of 100% for each target (Table 4). The IC showed a positivity rate of 99.17% (119/120). A summary of diagnostic performance based on confusion matrix analysis is presented in Table 5. For RSV A detection, the multiplex RSV A/B/IC LAMP assay correctly identified 84 of 91 RSV A positive specimens, yielding a sensitivity of 92.31% (95% CI, 85.0–96.8%), a specificity of 100% (95% CI, 98.3–100%), a PPV of 100%, an NPV of 96.87%, and an overall accuracy of 97.73%. For RSV B detection, 96 of 97 RSV B positive specimens were correctly identified, with a sensitivity of 98.97% (95% CI, 94.0–99.8%), a specificity of 100% (95% CI, 98.3–100%), a PPV of 100%, an NPV of 99.53%, and an accuracy of 99.68%. No false-positive results were observed for either RSV A or RSV B across all tested samples (n = 308), confirming the high specificity of the assay. Cohen’s κ coefficients were 0.944 for RSV A and 0.992 for RSV B, indicating almost perfect agreement between the multiplex RT-LAMP assay and the reference diagnosis (Allplex Respiratory Panel 1 assay).

**Table 5:** Diagnostic Performance of the multiplex RSV A/B/IC LAMP assay.

Multiplex RSV A/B/IC LAMP assay										
Target	TP	FN	TN	FP	Sensitivity (95% CI)	Specificity (95% CI)	PPV	NPV	Accuracy	Cohen’s κ
RSV A	84	7	217	0	92.31% (85.0–96.8)	100% (98.3–100)	100%	96.87%	97.73%	0.944
RSV B	96	1	211	0	98.97% (94.0–99.8)	100% (98.3–100)	100%	99.53%	99.68%	0.992

TP, true positive; FN, false negative; TN, true negative; FP, false positive; PPV, positive predictive value; NPV, negative predictive value. Accuracy = (TP + TN)/ total samples. For RSV A, TN includes RSV B positive specimens (n = 97) and RSV negative specimens (n = 120). For RSV B, TN includes RSV A positive specimens (n = 91) and RSV negative specimens (n = 120). Cohen’s κ values above 0.80 indicate almost perfect agreement between the two assays.

### Cross-reactivity of the multiplex RSV A/B/IC LAMP assay

The multiplex RSV A/B/IC LAMP assay was tested against a panel of 17 common respiratory pathogens, including 12 clinical respiratory viruses, 2 bacterial clinical specimens (*M. pneumoniae* and *B. pertussis*), and 3 cultured bacterial isolates (*S. pneumoniae, S. aureus,* and *K. pneumoniae*), all tested in triplicate. Across all tested non-RSV respiratory viruses, no amplification signals were observed in the RSV A (FAM) or RSV B (Cy5) channels, indicating the absence of cross-reactivity with the RSV-specific primers and probes. In contrast, the internal control (IC) channel consistently produced amplification signals for all clinical samples, confirming successful reaction performance and assay validity. The three cultured bacterial isolates (*S. pneumoniae, S. aureus,* and *K. pneumoniae*) did not yield IC signals, as these specimens lacked the human genomic background required for IC amplification. These results demonstrate that the multiplex RSV A/B/IC LAMP assay specifically detects RSV A and RSV B targets without nonspecific amplification from other respiratory pathogens (Table 6).

**Table 6:** Cross-reactivity testing of the multiplex RSV A/B/IC LAMP assay with common bacterial and viral respiratory pathogens.

Pathogens	Multiplex RSV A/B/IC LAMP assay (Tt ± SD)		
	RSV A (FAM)	RSV B (Cy5)	IC (Hex)
*Human coronavirus 229E*	N/D	N/D	14.13 ± 0.12
*Human coronavirus NL63*	N/D	N/D	13.79 ± 1.11
*Human coronavirus OC43*	N/D	N/D	14.18 ± 1.76
*Influenza A virus (H1N1 subtype)*	N/D	N/D	16.82 ± 0.21
*Influenza A virus (H3N2 subtype)*	N/D	N/D	16.91 ± 0.29
*Influenza B virus*	N/D	N/D	15.42 ± 0.14
*Human enterovirus*	N/D	N/D	16.50 ± 0.55
*Human adenovirus*	N/D	N/D	18.52 ± 2.63
*Human parainfluenza virus type 1*	N/D	N/D	19.34 ± 0.90
*Human metapneumovirus*	N/D	N/D	18.17 ± 1.39
*Human bocavirus*	N/D	N/D	17.31 ± 0.15
*Human rhinovirus*	N/D	N/D	16.13 ± 0.98
*Mycoplasma pneumoniae*	N/D	N/D	14.67 ± 0.52
*Bordetella pertussis*	N/D	N/D	18.40 ± 2.19
*Streptococcus pneumoniae*	N/D	N/D	N/D
*Staphylococcus aureus*	N/D	N/D	N/D
*Klebsiella pneumoniae*	N/D	N/D	N/D

Tt, time to threshold (minutes); SD, standard deviation; N/D, not detected.

## Discussion

Accurate differentiation between respiratory syncytial virus (RSV) subtypes A and B is clinically and epidemiologically important. With advances in isothermal amplification–based molecular diagnostics, a wide range of multiplex LAMP assays have been developed, and RT-LAMP–based approaches for RSV detection have been actively explored. However, RSV A and RSV B share a high degree of sequence similarity, which presents specific challenges for achieving reliable subtype-specific amplification and signal interpretation within a single isothermal reaction. As a result, most previously reported RSV RT-LAMP assays have focused on collective RSV detection by combining primer sets targeting RSV A and RSV B within a single reaction, typically relying on non–sequence-specific signal generation methods such as intercalating dyes or colorimetric indicators. While these approaches are effective for rapid RSV screening, they do not provide subtype-specific discrimination between RSV A and RSV B in a single-reaction format.

In this study, we developed and clinically evaluated a multiplex RSV A/B/IC RT-LAMP assay capable of subtype-specific detection of RSV A and RSV B within a single isothermal reaction. By integrating probe-based fluorescence detection into a single-tube multiplex RT-LAMP format, the assay enables simultaneous subtype discrimination while incorporating an internal control to verify reaction validity. Clinical evaluation using nasopharyngeal specimens demonstrated reliable subtype-dependent detection with high specificity and no cross-reactivity against a broad panel of respiratory pathogens, supporting the feasibility of probe-assisted multiplex RT-LAMP for subtype-resolved RSV diagnostics. During assay development, we initially sought to implement the multiplex RT-LAMP assay using hybridization-based TaqMan-style probes (HyTaq probes) for all targets. Under singleplex conditions, RSV A, RSV B, and the internal control were reliably detected using HyTaq probes. However, upon transition to the multiplex format, a reproducible attenuation of the RSV A signal was observed, despite stable amplification of RSV B and the internal control (S1 Fig). To address this issue, an assimilating probe was applied for RSV A in the multiplex assay, resulting in improved signal stability and enabling balanced and reproducible signal generation across all targets, including RSV A, RSV B, and the internal control, in a single-tube format (S1 Fig). These findings suggest that the use of different probe chemistries may provide an additional option for improving signal stability in multiplex isothermal amplification assays.

The analytical LOD of the multiplex RSV A/B/IC LAMP assay was 10⁴ copies/reaction for both RSV A and RSV B plasmid standards, which was higher than the LOD values reported in some previous RT-LAMP studies for RSV detection [11,19]. Reported analytical LOD values for RT-LAMP assays vary considerably across studies because they are influenced by differences in assay design, reference materials, and evaluation methods; therefore, direct comparison of published LOD values should be interpreted with caution. To provide a clinically relevant comparison, we directly evaluated the analytical sensitivity of the multiplex RT-LAMP assay against a commercial RT-qPCR assay using serially diluted RSV-positive clinical specimens under identical experimental conditions. The analytical LOD of the RT-LAMP assay was approximately one log higher than that of RT-qPCR when directly compared using the same clinical specimens under identical conditions. This direct, side-by-side comparison provides a more accurate and clinically relevant assessment of relative sensitivity than indirect comparisons across studies using different reference materials and protocols.

Clinical evaluation using nasopharyngeal specimens demonstrated that, based on the reference diagnosis, the multiplex RSV A/B/IC RT-LAMP assay showed a sensitivity of 92.31% for RSV A and 98.97% for RSV B, with a specificity of 100%. Under the same reference standard, the Allplex SARS-CoV-2/FluA/FluB/RSV Assay (Seegene, Seoul, Republic of Korea) showed sensitivities of 94.51% for RSV A and 98.97% for RSV B, with a specificity of 100% for both targets. These findings indicate that the multiplex RT-LAMP assay achieved clinical performance comparable to that of the commercial RT-PCR assay, with identical sensitivity for RSV B and only a small difference for RSV A. To further investigate the small difference in sensitivity observed for RSV A, the seven RT-LAMP false-negative specimens were analyzed in relation to the comparative RT-PCR results and Ct values. Among these specimens, three were also negative by the comparative Allplex SARS-CoV-2/FluA/FluB/RSV Assay despite being positive according to the original reference diagnosis. Of the remaining four specimens, three exhibited relatively high Ct values (28.48, 29.03, and 30.69), whereas only one specimen had a lower Ct value (21.50). Overall, six of the seven false-negative specimens were either undetectable by the comparative RT-PCR assay or exhibited relatively low viral RNA levels. These findings suggest that the slightly lower analytical sensitivity of the RT-LAMP assay primarily resulted in false-negative results among specimens with low viral loads, while having only a limited impact on overall clinical diagnostic performance.

When benchmarked against antigen-based rapid diagnostic tests (RDTs) reported in the literature, the clinical sensitivity of the present RT-LAMP assay is substantially higher. Previous studies have shown that commercially available RSV antigen RDTs typically exhibit sensitivities of approximately 60–75% relative to RT-qPCR, despite maintaining high specificity, with reduced performance in specimens with lower viral loads [20,21]. Similarly, comparative evaluations of RSV A/B antigen tests have reported sensitivities predominantly in the 60% range for both subtypes, while specificities generally exceed 95% [22].

In this context, the multiplex RSV A/B/IC RT-LAMP assay offers a practical advantage by combining high diagnostic sensitivity comparable to RT-qPCR with the operational simplicity and rapid turnaround of isothermal amplification. Rather than serving as a replacement for RT-qPCR, the assay may function as a complementary molecular diagnostic tool that overcomes the sensitivity limitations of antigen-based testing in point-of-care or decentralized settings. The ability to discriminate RSV A and RSV B within a single reaction, without the need for complex thermal cycling instrumentation, further enhances its clinical utility. This study has several limitations. First, all clinical specimens were collected from a single tertiary care center, which may limit the generalizability of the findings to other geographic regions or patient populations with different RSV subtype distributions. Multi-center prospective validation would therefore be necessary to confirm the broader applicability of the assay across diverse clinical settings. Second, the retrospective study design and the use of residual clinical specimens precluded prospective validation under routine diagnostic conditions and may introduce selection bias; therefore, prospective validation is required to confirm real-world performance. Third, all LAMP reactions were performed using a CFX96 real-time PCR detection system, which does not reflect true point-of-care conditions; evaluation using portable isothermal fluorescence readers would be necessary to fully validate the operational applicability of the assay in decentralized settings.

In conclusion, this study demonstrates the clinical feasibility of a probe-based multiplex RT-LAMP assay for reliable subtype-specific detection of RSV A and RSV B under isothermal conditions. By enabling single-reaction subtype resolution with high clinical concordance and operational simplicity, this approach extends the diagnostic utility of RT-LAMP beyond basic pathogen detection and provides a practical framework for the development of multiplex isothermal assays targeting respiratory viruses.

### Institutional review board statement

The study was conducted in accordance with the Declaration of Helsinki and approved by the Institutional Review Board of Korea University Guro Hospital (IRB No. 2019GR0055). The study protocol received an extension approval on 8 December 2025 to permit continued analysis and publication of the retrospective specimens.

### Informed consent statement

The requirement for informed consent was waived due to the use of anonymized residual clinical specimens and the minimal risk to participants. All samples were anonymized prior to analysis, and no identifiable information was used.

## Supporting information

S1 Fig Evaluation of RSV A signal attenuation in multiplex conditions using HyTaq probes and its recovery using an assimilating probe. Real-time amplification curves were obtained using RSV A, RSV B, and ACTB plasmid templates (10⁷ copies/reaction). (A) Singleplex conditions with HyTaq probes for all targets, demonstrating reliable detection of each target. (B) Multiplex conditions with HyTaq probes for all targets, showing reproducible attenuation of the RSV A signal. (C) Multiplex conditions with an assimilating probe for RSV A and HyTaq probes for RSV B and ACTB, demonstrating recovery of RSV A signal. Fluorescence signals were monitored in the FAM channel (green) for RSV A, the Cy5 channel (purple) for RSV B, and the HEX channel (blue) for ACTB.
(DOCX)

S2 Dataset Raw data underlying the manuscript. This file contains the Ct and RFU values obtained using the multiplex RSV A/B/IC LAMP assay for RSV A/B-positive and negative clinical samples, limit of detection testing, and cross-reactivity testing, along with the corresponding results from the Seegene RT-PCR kit.
(XLSX)
