# Supplementary material for: A multiplex dual-probe RT-LAMP assay for rapid subtype-specific detection of respiratory syncytial virus A and B
Source: PLoS One. 2026 Jul 31;21(7):e0354914. doi: 10.1371/journal.pone.0354914 (PMC13426929; doi:10.1371/journal.pone.0354914)
Supplement: S1 Fig — Real-time amplification curves were obtained using RSV A, RSV B, and ACTB plasmid templates (10⁷ copies/reaction). (A) Singleplex conditions with HyTaq probes for all targets, demonstrating reliable detection of each target. (B) Multiplex conditions with HyTaq probes for all targets, showing reproducible attenuation of the RSV A signal. (C) Multiplex conditions with an assimilating probe for RSV A and HyTaq probes for RSV B and ACTB, demonstrating recovery of RSV A signal. Fluorescence signals were monitored in the FAM channel (green) for RSV A, the Cy5 channel (purple) for RSV B, and the HEX channel (blue) for ACTB. (DOCX) [file pone.0354914.s001.docx]

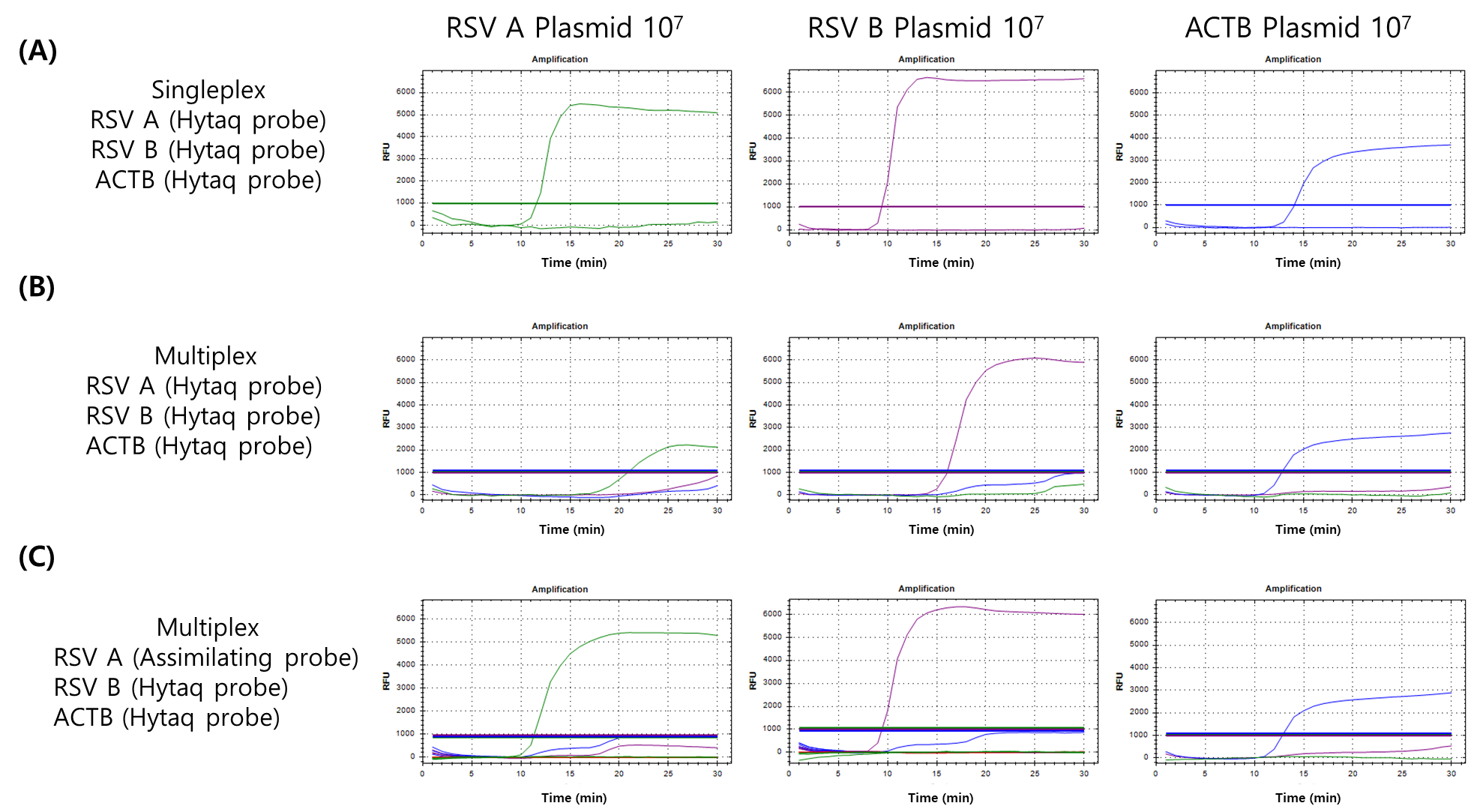


**S1 Fig. Evaluation of RSV A signal attenuation in multiplex conditions using HyTaq probes and its recovery using an assimilating probe.**

Real-time amplification curves were obtained using RSV A, RSV B, and ACTB plasmid templates (10⁷ copies/reaction). (A) Singleplex conditions with HyTaq probes for all targets, demonstrating reliable detection of each target. (B) Multiplex conditions with HyTaq probes for all targets, showing reproducible attenuation of the RSV A signal. (C) Multiplex conditions with an assimilating probe for RSV A and HyTaq probes for RSV B and ACTB, demonstrating recovery of RSV A signal. Fluorescence signals were monitored in the FAM channel (green) for RSV A, the Cy5 channel (purple) for RSV B, and the HEX channel (blue) for ACTB.
