# Supplementary material for: A multiplex dual-probe RT-LAMP assay for rapid subtype-specific detection of respiratory syncytial virus A and B
Source: PLoS One. 2026 Jul 31;21(7):e0354914. doi: 10.1371/journal.pone.0354914 (PMC13426929; doi:10.1371/journal.pone.0354914)
Supplement: Attachment — Submitted filename: Dear Authors.docx [file pone.0354914.s003.docx]

Dear Authors,

The manuscript has a fairly good scientific integrity and structure. Despite these, several significant and minor changes are needed to enhance scientific rigor, clarity and interpretation.

1. Overall Assessment

The manuscript describes the development and clinical testing of an innovative multiplex dual-probe RT-LAMP test which allows for simultaneous and subtype-specific detection of both RSV A and RSV B in a single reaction, using an IAC (internal amplification control). The study is relevant to an important diagnostic need especially in decentralized and point-of-care testing environments. The manuscript has a good writing style and is methodologically sound and clinically relevant.

2. Novelty and Significance

The most significant feature of this study is the novel design and use of two different probe chemistries (assimilating probe for RSV A and probes for RSV B and internal control) for reliable discrimination of the two different subtypes in a multiplex RT-LAMP format. According to the authors’ knowledge, it is a significant improvement on previously published RSV RT-LAMP assays that mostly used non-specific detection methods. The work can have implications for molecular epidemiological surveillance and near patient diagnostics.

3. Methodological Evaluation

The experimental design is suitable and analytical sensitivity testing, crossreactivity and clinical validation with a relatively large number of stored nasopharyngeal specimens were performed. An internal control provides robustness to the assay. There are a few things that need to be clarified however:

The limit of detection was reported as 10⁴ copies/reaction, which seems fairly low by today's standards for many RT-LAMP platforms. The authors should include a discussion about factors that may have caused the difference in analytical sensitivity.

All statistical analyses were carried out only with the help of Microsoft Excel. The use of statistical software which focuses specifically on this topic and the use of agreement statistics, such as Cohen's kappa, would increase the analytical rigor.

Only 12 respiratory viruses were tested for cross reactivity. More specificity data would be available if there were additional clinically relevant respiratory pathogens included, especially bacterial pathogens.

4. Results and Interpretation

Clinical performance was favourable and high sensitivity was observed (92.31% for RSV A, 98.97% for RSV B and 100% specificity for both). The results confirm that multiplex probe based RT-LAMP is feasible for the detection of RSV subtypes. However, the lower sensitivity noted for RSV A requires additional consideration as a result of the seven false-negative specimens and the viral load/CT data for these samples.

5. Limitations

The authors have recognized the limitations of this study such as the single centre design and real time PCR platform. Further potential multi-center studies could be useful for determining generalizability and in the real world.

6. Recommendation

Overall, this manuscript reports a clinically relevant and technically innovative approach to diagnostic testing that has potential relevance to decentralized RSV testing. The study should be published upon minor revision of methodological aspects clarifications and extended discussions as suggested above.

Recommendation: Minor Revision
