# Supplementary material for: A multiplex dual-probe RT-LAMP assay for rapid subtype-specific detection of respiratory syncytial virus A and B
Source: PLoS One. 2026 Jul 31;21(7):e0354914. doi: 10.1371/journal.pone.0354914 (PMC13426929; doi:10.1371/journal.pone.0354914)
Supplement: Attachment — Submitted filename: Response to Reviewers.docx [file pone.0354914.s005.docx]

**Response to Reviewer #1**

**Comment 1:** Lines 111-141 Inasmuch as this section is vital, it is too long. I suggest you break it down into two or three, and include sections for ethic statement/approval and cross-reactivity.

**Response:** We thank the reviewer for this helpful suggestion. In accordance with the reviewer's recommendation, we reorganized this section by dividing it into separate subsections and added dedicated sections for the ethical statement and cross-reactivity testing. The revised manuscript now includes the following subsections:

Clinical samples and DNA extraction **(Lines 115–131)**

Ethical statement **(Lines 132–138)**

Cross-reactivity testing **(Lines 218–230)**

**Comment 2:** Line 323 Capture cross-reactivity under methods, and report the outcome under the results. Do not repeat method/explanation under results.

**Response:** We thank the reviewer for this helpful comment. In accordance with the reviewer's suggestion, we moved the description of the cross-reactivity testing procedure to the Methods section and revised the Results section to report only the experimental findings without repeating the methodology. The relevant sections have been revised as follows:

**“Cross-reactivity Testing**

To evaluate cross-reactivity, a panel of respiratory pathogen–positive clinical specimens was included. The panel comprised one sample positive for each of the following 12 pathogens: human coronavirus 229E, coronavirus NL63, coronavirus OC43, influenza A (H1N1), influenza A (H3N2), influenza B, human enterovirus (HEV), adenovirus (AdV), parainfluenza virus (PIV), human metapneumovirus (hMPV), human bocavirus (HBoV), and human rhinovirus (HRV). These pathogens were confirmed by routine molecular diagnostic testing performed at the Department of Laboratory Medicine, Korea University Guro Hospital, independently of the LAMP assay. Each sample was tested in triplicate using the multiplex RSV A/B/IC LAMP assay under the same conditions described above.” **(Line 218-230)**

“**Cross-reactivity of the multiplex RSV A/B/IC LAMP assay**

The multiplex RSV A/B/IC LAMP assay was tested against a panel of 17 common respiratory pathogens, including 12 clinical respiratory viruses, 2 bacterial clinical specimens (*M. pneumoniae* and *B. pertussis*), and 3 cultured bacterial isolates (*S. pneumoniae, S. aureus,* and *K. pneumoniae*), all tested in triplicate. Across all tested non-RSV respiratory viruses and bacteria, no amplification signals were observed in the RSV A (FAM) or RSV B (Cy5) channels, indicating the absence of cross-reactivity with the RSV-specific primers and probes. In contrast, the internal control (IC) channel consistently produced amplification signals for all clinical samples, confirming successful reaction performance and assay validity. The three cultured bacterial isolates (S. pneumoniae, S. aureus, and K. pneumoniae) did not yield IC signals, as these specimens lacked the human genomic background required for IC amplification. These results demonstrate that the multiplex RSV A/B/IC LAMP assay specifically detects RSV A and RSV B targets without nonspecific amplification from other respiratory pathogens (Table 6).” **(Line 341-354)**

**Response to Reviewer #2**

The manuscript has a fairly good scientific integrity and structure. Despite these, several significant and minor changes are needed to enhance scientific rigor, clarity and interpretation.

**Response:** We thank Reviewer #2 for the positive assessment of our manuscript.

**1. Overall Assessment:** The manuscript describes the development and clinical testing of an innovative multiplex dual-probe RT-LAMP test which allows for simultaneous and subtype-specific detection of both RSV A and RSV B in a single reaction, using an IAC (internal amplification control). The study is relevant to an important diagnostic need especially in decentralized and point-of-care testing environments. The manuscript has a good writing style and is methodologically sound and clinically relevant.

**Response:** We thank Reviewer #2 for the positive assessment of our manuscript.

**2. Novelty and Significance:** The most significant feature of this study is the novel design and use of two different probe chemistries (assimilating probe for RSV A and probes for RSV B and internal control) for reliable discrimination of the two different subtypes in a multiplex RT-LAMP format. According to the authors’ knowledge, it is a significant improvement on previously published RSV RT-LAMP assays that mostly used non-specific detection methods. The work can have implications for molecular epidemiological surveillance and near patient diagnostics.

**Response:** We thank Reviewer #2 for recognizing the novelty of the dual-probe chemistry strategy and its potential implications for molecular epidemiological surveillance and point-of-care diagnostics.

**3. Methodological Evaluation:** The experimental design is suitable and analytical sensitivity testing, crossreactivity and clinical validation with a relatively large number of stored nasopharyngeal specimens were performed. An internal control provides robustness to the assay. There are a few things that need to be clarified however:

The limit of detection was reported as 10⁴ copies/reaction, which seems fairly low by today's standards for many RT-LAMP platforms. The authors should include a discussion about factors that may have caused the difference in analytical sensitivity.

**Response:** We thank the reviewer for this valuable comment. We agree that analytical sensitivity is an important performance characteristic. However, reported LOD values for RT-LAMP assays vary considerably among studies because they are influenced by differences in assay design, reference materials, and evaluation methods, making direct comparison difficult. To provide a more clinically relevant assessment, we additionally compared the analytical sensitivity of our multiplex RT-LAMP assay with that of a commercial RT-qPCR assay using serially diluted RSV-positive clinical specimens. We have revised the Discussion accordingly.

“The analytical LOD of the multiplex RSV A/B/IC LAMP assay was 10⁴ copies/reaction for both RSV A and RSV B plasmid standards, which was higher than the LOD values reported in some previous RT-LAMP studies for RSV detection [11, 19]. Reported analytical LOD values for RT-LAMP assays vary considerably across studies because they are influenced by differences in assay design, reference materials, and evaluation methods; therefore, direct comparison of published LOD values should be interpreted with caution. To provide a clinically relevant comparison, we directly evaluated the analytical sensitivity of the multiplex RT-LAMP assay against a commercial RT-qPCR assay using serially diluted RSV-positive clinical specimens under identical experimental conditions. The analytical LOD of the RT-LAMP assay was approximately one log higher than that of RT-qPCR when directly compared using the same clinical specimens under identical conditions. This direct, side-by-side comparison provides a more accurate and clinically relevant assessment of relative sensitivity than indirect comparisons across studies using different reference materials and protocols.” **(Line 392-404)**

All statistical analyses were carried out only with the help of Microsoft Excel. The use of statistical software which focuses specifically on this topic and the use of agreement statistics, such as Cohen's kappa, would increase the analytical rigor.

**Response:** We thank the reviewer for this valuable suggestion. We agree that agreement statistics provide a more rigorous assessment of diagnostic performance. Accordingly, we calculated Cohen's κ coefficient to assess the agreement between the multiplex RT-LAMP assay and the reference diagnosis. Cohen's κ coefficients were 0.944 for RSV A and 0.992 for RSV B, indicating almost perfect agreement between the two methods. These results have been incorporated into the Materials and Methods **(Line 238-242)**, Results **(Line 324-326)**, and Table 5 of the revised manuscript.

Only 12 respiratory viruses were tested for cross reactivity. More specificity data would be available if there were additional clinically relevant respiratory pathogens included, especially bacterial pathogens.

**Response:** We thank the reviewer for this suggestion. We agree that a broader cross-reactivity panel would strengthen the specificity data. Accordingly, we have expanded the panel to include five additional clinically relevant bacterial respiratory pathogens (Mycoplasma pneumoniae, Bordetella pertussis, Streptococcus pneumoniae, Staphylococcus aureus, and Klebsiella pneumoniae). No amplification was observed in the RSV A (FAM) or RSV B (Cy5) channels for any of the additionally tested pathogens, further confirming the specificity of the assay. The updated cross-reactivity panel (17 pathogens in total) and corresponding results have been incorporated into the revised Materials and Methods **(Line 224-228)**, Results **(Line 342-351)**, and Table 6.

**4. Results and Interpretation:** Clinical performance was favourable and high sensitivity was observed (92.31% for RSV A, 98.97% for RSV B and 100% specificity for both). The results confirm that multiplex probe based RT-LAMP is feasible for the detection of RSV subtypes. However, the lower sensitivity noted for RSV A requires additional consideration as a result of the seven false-negative specimens and the viral load/CT data for these samples.

**Response:** We thank the reviewer for this valuable comment. In response to this suggestion, we further analyzed the seven RSV A false-negative specimens by reviewing the comparative RT-PCR results and Ct values and incorporated the findings into the Discussion. The Discussion has been revised as follows:

“These findings indicate that the multiplex RT-LAMP assay achieved clinical performance comparable to that of the commercial RT-PCR assay, with identical sensitivity for RSV B and only a small difference for RSV A. To further investigate the small difference in sensitivity observed for RSV A, the seven RT-LAMP false-negative specimens were analyzed in relation to the comparative RT-PCR results and Ct values. Among these specimens, three were also negative by the comparative Allplex SARS-CoV-2/FluA/FluB/RSV Assay despite being positive according to the original reference diagnosis. Of the remaining four specimens, three exhibited relatively high Ct values (28.48, 29.03, and 30.69), whereas only one specimen had a lower Ct value (21.50). Overall, six of the seven false-negative specimens were either undetectable by the comparative RT-PCR assay or exhibited relatively low viral RNA levels. These findings suggest that the slightly lower analytical sensitivity of the RT-LAMP assay primarily resulted in false-negative results among specimens with low viral loads, while having only a limited impact on overall clinical diagnostic performance.” **(Line 410-422)**

**5. Limitations:** The authors have recognized the limitations of this study such as the single centre design and real time PCR platform. Further potential multi-center studies could be useful for determining generalizability and in the real world.

**Response:** We thank the reviewer for this valuable suggestion. We agree that additional multi-center studies would strengthen the generalizability of our findings. Accordingly, we have revised the Discussion to acknowledge this limitation and have added the following statement:

“Multi-center prospective validation would therefore be necessary to confirm the broader applicability of the assay across diverse clinical settings.” **(Line 439-441)**

6. Recommendation

Overall, this manuscript reports a clinically relevant and technically innovative approach to diagnostic testing that has potential relevance to decentralized RSV testing. The study should be published upon minor revision of methodological aspects clarifications and extended discussions as suggested above.
