# Supplementary material for: A multiplex dual-probe RT-LAMP assay for rapid subtype-specific detection of respiratory syncytial virus A and B
Source: PLoS One. 2026 Jul 31;21(7):e0354914. doi: 10.1371/journal.pone.0354914 (PMC13426929; doi:10.1371/journal.pone.0354914)
Supplement: Peer review history

# Peer review: Decision Letter 0

**DOI:** 10.1371/journal.pone.0354914.r001

**Authors:** Haitham Amer

18 Jun 2026

PONE-D-26-19650

A Multiplex Dual-Probe RT-LAMP Assay for Rapid Subtype-Specific Detection of Respiratory Syncytial Virus A and B

PLOS One

Dear Dr.  Jang,

Thank you for submitting your manuscript to PLOS ONE. After careful consideration, we feel that it has merit but does not fully meet PLOS ONE’s publication criteria as it currently stands. Therefore, we invite you to submit a revised version of the manuscript that addresses the points raised during the review process.

Please submit your revised manuscript by Aug 02 2026 11:59PM. If you will need more time than this to complete your revisions, please reply to this message or contact the journal office at plosone@plos.org. When you're ready to submit your revision, log on to https://www.editorialmanager.com/pone/ and select the 'Submissions Needing Revision' folder to locate your manuscript file.

Please include the following items when submitting your revised manuscript:

- A letter that responds to each point raised by the academic editor and reviewer(s). You should upload this letter as a separate file labeled 'Response to Reviewers'.
- A marked-up copy of your manuscript that highlights changes made to the original version. You should upload this as a separate file labeled 'Revised Manuscript with Track Changes'.
- An unmarked version of your revised paper without tracked changes. You should upload this as a separate file labeled 'Manuscript'.

If you would like to make changes to your financial disclosure, please include your updated statement in your cover letter. Guidelines for resubmitting your figure files are available below the reviewer comments at the end of this letter.

If applicable, we recommend that you deposit your laboratory protocols in protocols.io to enhance the reproducibility of your results. Protocols.io assigns your protocol its own identifier (DOI) so that it can be cited independently in the future. For instructions see: https://journals.plos.org/plosone/s/submission-guidelines#loc-laboratory-protocols. Additionally, PLOS ONE offers an option for publishing peer-reviewed Lab Protocol articles, which describe protocols hosted on protocols.io. Read more information on sharing protocols at https://plos.org/protocols?utm_medium=editorial-email&utm_source=authorletters&utm_campaign=protocols.

As the corresponding author, your ORCID iD is verified in the submission system and will appear in the published article. PLOS supports the use of ORCID, and we encourage all coauthors to register for an ORCID iD and use it as well. Please encourage your coauthors to verify their ORCID iD within the submission system before final acceptance, as unverified ORCID iDs will not appear in the published article. *Only*  the individual author can complete the verification step; PLOS staff *cannot*  verify ORCID iDs on behalf of authors.

We look forward to receiving your revised manuscript.

Kind regards,

Haitham Mohamed Amer, PhD

Academic Editor

PLOS One

Journal Requirements:

When submitting your revision, we need you to address these additional requirements.

1.Please ensure that your manuscript meets PLOS ONE's style requirements, including those for file naming. The PLOS ONE style templates can be found at

https://journals.plos.org/plosone/s/file?id=wjVg/PLOSOne_formatting_sample_main_body.pdf and

https://journals.plos.org/plosone/s/file?id=ba62/PLOSOne_formatting_sample_title_authors_affiliations.pdf

2. Thank you for stating the following financial disclosure:

“This work was supported by a grant from the Korea Health Technology R&D Project through the Korea Health Industry Development Institute (KHIDI), funded by the Ministry of Health and Welfare, Republic of Korea (grant number: HR20C0021).”

Please state what role the funders took in the study.  If the funders had no role, please state: "The funders had no role in study design, data collection and analysis, decision to publish, or preparation of the manuscript."

If this statement is not correct you must amend it as needed.

Please include this amended Role of Funder statement in your cover letter; we will change the online submission form on your behalf.

3. Please note that funding information should not appear in any section or other areas of your manuscript. We will only publish funding information present in the Funding Statement section of the online submission form. Please remove any funding-related text from the manuscript.

4. Your ethics statement should only appear in the Methods section of your manuscript. If your ethics statement is written in any section besides the Methods, please move it to the Methods section and delete it from any other section. Please ensure that your ethics statement is included in your manuscript, as the ethics statement entered into the online submission form will not be published alongside your manuscript.

5. Please include captions for your Supporting Information files at the end of your manuscript, and update any in-text citations to match accordingly. Please see our Supporting Information guidelines for more information: http://journals.plos.org/plosone/s/supporting-information.

6. If the reviewer comments include a recommendation to cite specific previously published works, please review and evaluate these publications to determine whether they are relevant and should be cited. There is no requirement to cite these works unless the editor has indicated otherwise.

7. Please review your reference list to ensure that it is complete and correct. If you have cited papers that have been retracted, please include the rationale for doing so in the manuscript text, or remove these references and replace them with relevant current references. Any changes to the reference list should be mentioned in the rebuttal letter that accompanies your revised manuscript. If you need to cite a retracted article, indicate the article’s retracted status in the References list and also include a citation and full reference for the retraction notice.

Reviewer's Responses to Questions

**Comments to the Author**

1. Is the manuscript technically sound, and do the data support the conclusions?

The manuscript must describe a technically sound piece of scientific research with data that supports the conclusions. Experiments must have been conducted rigorously, with appropriate controls, replication, and sample sizes. The conclusions must be drawn appropriately based on the data presented.

Reviewer #1: Yes

Reviewer #2: Yes

**********

2. Has the statistical analysis been performed appropriately and rigorously?

Reviewer #1: Yes

Reviewer #2: Yes

**********

3. Have the authors made all data underlying the findings in their manuscript fully available?

The PLOS Data policy requires authors to make all data underlying the findings described in their manuscript fully available without restriction, with rare exception (please refer to the Data Availability Statement in the manuscript PDF file). The data should be provided as part of the manuscript or its supporting information, or deposited to a public repository. For example, in addition to summary statistics, the data points behind means, medians and variance measures should be available. If there are restrictions on publicly sharing data—e.g. participant privacy or use of data from a third party—those must be specified.

Reviewer #1: Yes

Reviewer #2: Yes

**********

4. Is the manuscript presented in an intelligible fashion and written in standard English?

PLOS ONE does not copyedit accepted manuscripts, so the language in submitted articles must be clear, correct, and unambiguous. Any typographical or grammatical errors should be corrected at revision, so please note any specific errors here.

Reviewer #1: Yes

Reviewer #2: Yes

**********

5. Review Comments to the Author

Please use the space provided to explain your answers to the questions above. You may also include additional comments for the author, including concerns about dual publication, research ethics, or publication ethics. (Please upload your review as an attachment if it exceeds 20,000 characters)

Reviewer #1: Lines 111-141 Inasmuch as this section is vital, it is too long. I suggest you break it down into two or three, and include sections for ethic statement/approval and cross-reactivity.

Line 323 Capture cross-reactivity under methods, and report the outcome under the results. Do not repeat method/explanation under results.

Reviewer #2: Dear Authors,

The manuscript has a fairly good scientific integrity and structure. Despite these, several significant and minor changes are needed to enhance scientific rigor, clarity and interpretation.

1. Overall Assessment

The manuscript describes the development and clinical testing of an innovative multiplex dual-probe RT-LAMP test which allows for simultaneous and subtype-specific detection of both RSV A and RSV B in a single reaction, using an IAC (internal amplification control). The study is relevant to an important diagnostic need especially in decentralized and point-of-care testing environments. The manuscript has a good writing style and is methodologically sound and clinically relevant.

2. Novelty and Significance

The most significant feature of this study is the novel design and use of two different probe chemistries (assimilating probe for RSV A and probes for RSV B and internal control) for reliable discrimination of the two different subtypes in a multiplex RT-LAMP format. According to the authors’ knowledge, it is a significant improvement on previously published RSV RT-LAMP assays that mostly used non-specific detection methods. The work can have implications for molecular epidemiological surveillance and near patient diagnostics.

3. Methodological Evaluation

The experimental design is suitable and analytical sensitivity testing, crossreactivity and clinical validation with a relatively large number of stored nasopharyngeal specimens were performed. An internal control provides robustness to the assay. There are a few things that need to be clarified however:

The limit of detection was reported as 10⁴ copies/reaction, which seems fairly low by today's standards for many RT-LAMP platforms. The authors should include a discussion about factors that may have caused the difference in analytical sensitivity.

All statistical analyses were carried out only with the help of Microsoft Excel. The use of statistical software which focuses specifically on this topic and the use of agreement statistics, such as Cohen's kappa, would increase the analytical rigor.

Only 12 respiratory viruses were tested for cross reactivity. More specificity data would be available if there were additional clinically relevant respiratory pathogens included, especially bacterial pathogens.

4. Results and Interpretation

Clinical performance was favourable and high sensitivity was observed (92.31% for RSV A, 98.97% for RSV B and 100% specificity for both). The results confirm that multiplex probe based RT-LAMP is feasible for the detection of RSV subtypes. However, the lower sensitivity noted for RSV A requires additional consideration as a result of the seven false-negative specimens and the viral load/CT data for these samples.

5. Limitations

The authors have recognized the limitations of this study such as the single centre design and real time PCR platform. Further potential multi-center studies could be useful for determining generalizability and in the real world.

6. Recommendation

Overall, this manuscript reports a clinically relevant and technically innovative approach to diagnostic testing that has potential relevance to decentralized RSV testing. The study should be published upon minor revision of methodological aspects clarifications and extended discussions as suggested above.

Recommendation: Minor Revision

**********

6. PLOS authors have the option to publish the peer review history of their article (what does this mean?). If published, this will include your full peer review and any attached files.

If you choose “no”, your identity will remain anonymous but your review may still be made public.

**Do you want your identity to be public for this peer review?**  For information about this choice, including consent withdrawal, please see our Privacy Policy.

Reviewer #1: No

Reviewer #2: **Yes:**  Dr. Bilal Tantry

**********

Attachment Submitted filename: Dear Authors.docx

# Author comment: Author response to Decision Letter 1

**DOI:** 10.1371/journal.pone.0354914.r002

30 Jun 2026

Response to Reviewer #1

Comment 1: Lines 111-141 Inasmuch as this section is vital, it is too long. I suggest you break it down into two or three, and include sections for ethic statement/approval and cross-reactivity.

Response: We thank the reviewer for this helpful suggestion. In accordance with the reviewer's recommendation, we reorganized this section by dividing it into separate subsections and added dedicated sections for the ethical statement and cross-reactivity testing. The revised manuscript now includes the following subsections:

Clinical samples and DNA extraction (Lines 115–131)

Ethical statement (Lines 132–138)

Cross-reactivity testing (Lines 218–230)

Comment 2: Line 323 Capture cross-reactivity under methods, and report the outcome under the results. Do not repeat method/explanation under results.

Response: We thank the reviewer for this helpful comment. In accordance with the reviewer's suggestion, we moved the description of the cross-reactivity testing procedure to the Methods section and revised the Results section to report only the experimental findings without repeating the methodology. The relevant sections have been revised as follows:

“Cross-reactivity Testing

To evaluate cross-reactivity, a panel of respiratory pathogen–positive clinical specimens was included. The panel comprised one sample positive for each of the following 12 pathogens: human coronavirus 229E, coronavirus NL63, coronavirus OC43, influenza A (H1N1), influenza A (H3N2), influenza B, human enterovirus (HEV), adenovirus (AdV), parainfluenza virus (PIV), human metapneumovirus (hMPV), human bocavirus (HBoV), and human rhinovirus (HRV). These pathogens were confirmed by routine molecular diagnostic testing performed at the Department of Laboratory Medicine, Korea University Guro Hospital, independently of the LAMP assay. Each sample was tested in triplicate using the multiplex RSV A/B/IC LAMP assay under the same conditions described above.” (Line 218-230)

“Cross-reactivity of the multiplex RSV A/B/IC LAMP assay

The multiplex RSV A/B/IC LAMP assay was tested against a panel of 17 common respiratory pathogens, including 12 clinical respiratory viruses, 2 bacterial clinical specimens (M. pneumoniae and B. pertussis), and 3 cultured bacterial isolates (S. pneumoniae, S. aureus, and K. pneumoniae), all tested in triplicate. Across all tested non-RSV respiratory viruses and bacteria, no amplification signals were observed in the RSV A (FAM) or RSV B (Cy5) channels, indicating the absence of cross-reactivity with the RSV-specific primers and probes. In contrast, the internal control (IC) channel consistently produced amplification signals for all clinical samples, confirming successful reaction performance and assay validity. The three cultured bacterial isolates (S. pneumoniae, S. aureus, and K. pneumoniae) did not yield IC signals, as these specimens lacked the human genomic background required for IC amplification. These results demonstrate that the multiplex RSV A/B/IC LAMP assay specifically detects RSV A and RSV B targets without nonspecific amplification from other respiratory pathogens (Table 6).” (Line 341-354)

Response to Reviewer #2

The manuscript has a fairly good scientific integrity and structure. Despite these, several significant and minor changes are needed to enhance scientific rigor, clarity and interpretation.

Response: We thank Reviewer #2 for the positive assessment of our manuscript.

1. Overall Assessment: The manuscript describes the development and clinical testing of an innovative multiplex dual-probe RT-LAMP test which allows for simultaneous and subtype-specific detection of both RSV A and RSV B in a single reaction, using an IAC (internal amplification control). The study is relevant to an important diagnostic need especially in decentralized and point-of-care testing environments. The manuscript has a good writing style and is methodologically sound and clinically relevant.

Response: We thank Reviewer #2 for the positive assessment of our manuscript.

2. Novelty and Significance: The most significant feature of this study is the novel design and use of two different probe chemistries (assimilating probe for RSV A and probes for RSV B and internal control) for reliable discrimination of the two different subtypes in a multiplex RT-LAMP format. According to the authors’ knowledge, it is a significant improvement on previously published RSV RT-LAMP assays that mostly used non-specific detection methods. The work can have implications for molecular epidemiological surveillance and near patient diagnostics.

Response: We thank Reviewer #2 for recognizing the novelty of the dual-probe chemistry strategy and its potential implications for molecular epidemiological surveillance and point-of-care diagnostics.

3. Methodological Evaluation: The experimental design is suitable and analytical sensitivity testing, crossreactivity and clinical validation with a relatively large number of stored nasopharyngeal specimens were performed. An internal control provides robustness to the assay. There are a few things that need to be clarified however:

The limit of detection was reported as 10⁴ copies/reaction, which seems fairly low by today's standards for many RT-LAMP platforms. The authors should include a discussion about factors that may have caused the difference in analytical sensitivity.

Response: We thank the reviewer for this valuable comment. We agree that analytical sensitivity is an important performance characteristic. However, reported LOD values for RT-LAMP assays vary considerably among studies because they are influenced by differences in assay design, reference materials, and evaluation methods, making direct comparison difficult. To provide a more clinically relevant assessment, we additionally compared the analytical sensitivity of our multiplex RT-LAMP assay with that of a commercial RT-qPCR assay using serially diluted RSV-positive clinical specimens. We have revised the Discussion accordingly.

“The analytical LOD of the multiplex RSV A/B/IC LAMP assay was 10⁴ copies/reaction for both RSV A and RSV B plasmid standards, which was higher than the LOD values reported in some previous RT-LAMP studies for RSV detection [11, 19]. Reported analytical LOD values for RT-LAMP assays vary considerably across studies because they are influenced by differences in assay design, reference materials, and evaluation methods; therefore, direct comparison of published LOD values should be interpreted with caution. To provide a clinically relevant comparison, we directly evaluated the analytical sensitivity of the multiplex RT-LAMP assay against a commercial RT-qPCR assay using serially diluted RSV-positive clinical specimens under identical experimental conditions. The analytical LOD of the RT-LAMP assay was approximately one log higher than that of RT-qPCR when directly compared using the same clinical specimens under identical conditions. This direct, side-by-side comparison provides a more accurate and clinically relevant assessment of relative sensitivity than indirect comparisons across studies using different reference materials and protocols.” (Line 392-404)

All statistical analyses were carried out only with the help of Microsoft Excel. The use of statistical software which focuses specifically on this topic and the use of agreement statistics, such as Cohen's kappa, would increase the analytical rigor.

Response: We thank the reviewer for this valuable suggestion. We agree that agreement statistics provide a more rigorous assessment of diagnostic performance. Accordingly, we calculated Cohen's κ coefficient to assess the agreement between the multiplex RT-LAMP assay and the reference diagnosis. Cohen's κ coefficients were 0.944 for RSV A and 0.992 for RSV B, indicating almost perfect agreement between the two methods. These results have been incorporated into the Materials and Methods (Line 238-242), Results (Line 324-326), and Table 5 of the revised manuscript.

Only 12 respiratory viruses were tested for cross reactivity. More specificity data would be available if there were additional clinically relevant respiratory pathogens included, especially bacterial pathogens.

Response: We thank the reviewer for this suggestion. We agree that a broader cross-reactivity panel would strengthen the specificity data. Accordingly, we have expanded the panel to include five additional clinically relevant bacterial respiratory pathogens (Mycoplasma pneumoniae, Bordetella pertussis, Streptococcus pneumoniae, Staphylococcus aureus, and Klebsiella pneumoniae). No amplification was observed in the RSV A (FAM) or RSV B (Cy5) channels for any of the additionally tested pathogens, further confirming the specificity of the assay. The updated cross-reactivity panel (17 pathogens in total) and corresponding results have been incorporated into the revised Materials and Methods (Line 224-228), Results (Line 342-351), and Table 6.

4. Results and Interpretation: Clinical performance was favourable and high sensitivity was observed (92.31% for RSV A, 98.97% for RSV B and 100% specificity for both). The results confirm that multiplex probe based RT-LAMP is feasible for the detection of RSV subtypes. However, the lower sensitivity noted for RSV A requires additional consideration as a result of the seven false-negative specimens and the viral load/CT data for these samples.

Response: We thank the reviewer for this valuable comment. In response to this suggestion, we further analyzed the seven RSV A false-negative specimens by reviewing the comparative RT-PCR results and Ct values and incorporated the findings into the Discussion. The Discussion has been revised as follows:

“These findings indicate that the multiplex RT-LAMP assay achieved clinical performance comparable to that of the commercial RT-PCR assay, with identical sensitivity for RSV B and only a small difference for RSV A. To further investigate the small difference in sensitivity observed for RSV A, the seven RT-LAMP false-negative specimens were analyzed in relation to the comparative RT-PCR results and Ct values. Among these specimens, three were also negative by the comparative Allplex SARS-CoV-2/FluA/FluB/RSV Assay despite being positive according to the original reference diagnosis. Of the remaining four specimens, three exhibited relatively high Ct values (28.48, 29.03, and 30.69), whereas only one specimen had a lower Ct value (21.50). Overall, six of the seven false-negative specimens were either undetectable by the comparative RT-PCR assay or exhibited relatively low viral RNA levels. These findings suggest that the slightly lower analytical sensitivity of the RT-LAMP assay primarily resulted in false-negative results among specimens with low viral loads, while having only a limited impact on overall clinical diagnostic performance.” (Line 410-422)

5. Limitations: The authors have recognized the limitations of this study such as the single centre design and real time PCR platform. Further potential multi-center studies could be useful for determining generalizability and in the real world.

Response: We thank the reviewer for this valuable suggestion. We agree that additional multi-center studies would strengthen the generalizability of our findings. Accordingly, we have revised the Discussion to acknowledge this limitation and have added the following statement:

“Multi-center prospective validation would therefore be necessary to confirm the broader applicability of the assay across diverse clinical settings.” (Line 439-441)

6. Recommendation

Overall, this manuscript reports a clinically relevant and technically innovative approach to diagnostic testing that has potential relevance to decentralized RSV testing. The study should be published upon minor revision of methodological aspects clarifications and extended discussions as suggested above.

Attachment Submitted filename: Response to Reviewers.docx

# Peer review: Decision Letter 1

**DOI:** 10.1371/journal.pone.0354914.r003

**Authors:** Haitham Amer

15 Jul 2026

A Multiplex Dual-Probe RT-LAMP Assay for Rapid Subtype-Specific Detection of Respiratory Syncytial Virus A and B

PONE-D-26-19650R1

Dear Dr. Jang,

We’re pleased to inform you that your manuscript has been judged scientifically suitable for publication and will be formally accepted for publication once it meets all outstanding technical requirements.

Within one week, you’ll receive an e-mail detailing the required amendments. When these have been addressed, you’ll receive a formal acceptance letter and your manuscript will be scheduled for publication.

An invoice will be generated when your article is formally accepted. Please note, if your institution has a publishing partnership with PLOS and your article meets the relevant criteria, all or part of your publication costs will be covered. Please make sure your user information is up-to-date by logging into Editorial Manager at Editorial Manager® and clicking the ‘Update My Information' link at the top of the page. For questions related to billing, please contact billing support.

If your institution or institutions have a press office, please notify them about your upcoming paper to help maximize its impact. If they’ll be preparing press materials, please inform our press team as soon as possible -- no later than 48 hours after receiving the formal acceptance. Your manuscript will remain under strict press embargo until 2 pm Eastern Time on the date of publication. For more information, please contact onepress@plos.org.

Kind regards,

Haitham Mohamed Amer, PhD

Academic Editor

PLOS One

Reviewers' comments:

Reviewer's Responses to Questions

**Comments to the Author**

1. If the authors have adequately addressed your comments raised in a previous round of review and you feel that this manuscript is now acceptable for publication, you may indicate that here to bypass the “Comments to the Author” section, enter your conflict of interest statement in the “Confidential to Editor” section, and submit your "Accept" recommendation.

Reviewer #1: All comments have been addressed

Reviewer #2: (No Response)

**********

2. Is the manuscript technically sound, and do the data support the conclusions?

The manuscript must describe a technically sound piece of scientific research with data that supports the conclusions. Experiments must have been conducted rigorously, with appropriate controls, replication, and sample sizes. The conclusions must be drawn appropriately based on the data presented.

Reviewer #1: Yes

Reviewer #2: Yes

**********

3. Has the statistical analysis been performed appropriately and rigorously?

Reviewer #1: Yes

Reviewer #2: Yes

**********

4. Have the authors made all data underlying the findings in their manuscript fully available?

The PLOS Data policy requires authors to make all data underlying the findings described in their manuscript fully available without restriction, with rare exception (please refer to the Data Availability Statement in the manuscript PDF file). The data should be provided as part of the manuscript or its supporting information, or deposited to a public repository. For example, in addition to summary statistics, the data points behind means, medians and variance measures should be available. If there are restrictions on publicly sharing data—e.g. participant privacy or use of data from a third party—those must be specified.

Reviewer #1: Yes

Reviewer #2: Yes

**********

5. Is the manuscript presented in an intelligible fashion and written in standard English?

PLOS ONE does not copyedit accepted manuscripts, so the language in submitted articles must be clear, correct, and unambiguous. Any typographical or grammatical errors should be corrected at revision, so please note any specific errors here.

Reviewer #1: Yes

Reviewer #2: Yes

**********

6. Review Comments to the Author

Please use the space provided to explain your answers to the questions above. You may also include additional comments for the author, including concerns about dual publication, research ethics, or publication ethics. (Please upload your review as an attachment if it exceeds 20,000 characters)

Reviewer #1: No additional comments at this point. Thanks to the authors for incorporating the comments to make the study more sound and robust.

Reviewer #2: Thank you very much, changes you have made are accepted in present form. the manuscript will be beneficial to scientific community.

kindest regards,

Bilal, Ph.D.

**********

7. PLOS authors have the option to publish the peer review history of their article (what does this mean?). If published, this will include your full peer review and any attached files.

If you choose “no”, your identity will remain anonymous but your review may still be made public.

**Do you want your identity to be public for this peer review?**  For information about this choice, including consent withdrawal, please see our Privacy Policy.

Reviewer #1: No

Reviewer #2: **Yes:**  Dr Bilal Tantry

**********

# Editor report: Acceptance letter

**DOI:** 10.1371/journal.pone.0354914.r004

**Authors:** Haitham Amer

PONE-D-26-19650R1

PLOS One

Dear Dr. Jang,

I'm pleased to inform you that your manuscript has been deemed suitable for publication in PLOS One. Congratulations! Your manuscript is now being handed over to our production team.

At this stage, our production department will prepare your paper for publication. This includes ensuring the following:

* All references, tables, and figures are properly cited

* All relevant supporting information is included in the manuscript submission,

* There are no issues that prevent the paper from being properly typeset

You will receive further instructions from the production team, including instructions on how to review your proof when it is ready. Please keep in mind that we are working through a large volume of accepted articles, so please give us a few days to review your paper and let you know the next and final steps.

Lastly, if your institution or institutions have a press office, please let them know about your upcoming paper now to help maximize its impact. If they'll be preparing press materials, please inform our press team within the next 48 hours. Your manuscript will remain under strict press embargo until 2 pm Eastern Time on the date of publication. For more information, please contact onepress@plos.org.

You will receive an invoice from PLOS for your publication fee after your manuscript has reached the completed accept phase. If you receive an email requesting payment before acceptance or for any other service, this may be a phishing scheme. Learn how to identify phishing emails and protect your accounts at https://explore.plos.org/phishing.

If we can help with anything else, please email us at customercare@plos.org.

Thank you for submitting your work to PLOS ONE and supporting open access.

Kind regards,

PLOS ONE Editorial Office Staff

on behalf of

Dr. Haitham Mohamed Amer

Academic Editor

PLOS One
